# Socioeconomic and Nutritional Factors Account for the Association of Gastric Cancer with Amerindian Ancestry in a Latin American Admixed Population

**DOI:** 10.1371/journal.pone.0041200

**Published:** 2012-08-03

**Authors:** Latife Pereira, Roxana Zamudio, Giordano Soares-Souza, Phabiola Herrera, Lilia Cabrera, Catherine C. Hooper, Jaime Cok, Juan M. Combe, Gloria Vargas, William A. Prado, Silvana Schneider, Fernanda Kehdy, Maira R. Rodrigues, Stephen J. Chanock, Douglas E. Berg, Robert H. Gilman, Eduardo Tarazona-Santos

**Affiliations:** 1 Departamento de Biologia Geral, Universidade Federal de Minas Gerais, Belo Horizonte, Brazil; 2 Asociación Benéfica PRISMA, Lima, Peru; 3 Laboratorios de Investigacion y Desarrollo, Facultad de Ciencias, Universidad Peruana Cayetano Heredia, Lima, Peru; 4 Departamento de Patología, Hospital Nacional Cayetano Heredia, Lima, Peru; 5 Departamento de Gastroenterologia, Instituto Nacional de Enfermedades Neopláscas, Lima, Peru; 6 Servicio de Gastroenterologia, Hospital Nacional Arzobispo Loayza, Lima, Peru; 7 Servicio de Gastroenterologia, Hospital Dos de Mayo, Lima, Peru; 8 Departamento de Estatística, Universidade Federal de Minas Gerais, Belo Horizonte, Brazil; 9 Laboratory of Translational Genomics of the Division of Cancer Epidemiology and Genetics, National Cancer Institute, National Institutes of Health, Gaithersburg, Maryland, United States of America; 10 Department of Molecular Microbiology, Washington University Medical School, St Louis, Missouri, United States of America; 11 Department of International Health, Bloomberg School of Public Health, Johns Hopkins University, Baltimore, Maryland, United States of America; National Institute of Environmental Health Sciences, United States of America

## Abstract

Gastric cancer is one of the most lethal types of cancer and its incidence varies worldwide, with the Andean region of South America showing high incidence rates. We evaluated the genetic structure of the population from Lima (Peru) and performed a case-control genetic association study to test the contribution of African, European, or Native American ancestry to risk for gastric cancer, controlling for the effect of non-genetic factors. A wide set of socioeconomic, dietary, and clinic information was collected for each participant in the study and ancestry was estimated based on 103 ancestry informative markers. Although the urban population from Lima is usually considered as mestizo (i.e., admixed from Africans, Europeans, and Native Americans), we observed a high fraction of Native American ancestry (78.4% for the cases and 74.6% for the controls) and a very low African ancestry (<5%). We determined that higher Native American individual ancestry is associated with gastric cancer, but socioeconomic factors associated both with gastric cancer and Native American ethnicity account for this association. Therefore, the high incidence of gastric cancer in Peru does not seem to be related to susceptibility alleles common in this population. Instead, our result suggests a predominant role for ethnic-associated socioeconomic factors and disparities in access to health services. Since Native Americans are a neglected group in genomic studies, we suggest that the population from Lima and other large cities from Western South America with high Native American ancestry background may be convenient targets for epidemiological studies focused on this ethnic group.

## Introduction

Gastric cancer is one of the most lethal types of cancer, accounting for approximately 800,000 deaths per year, but its incidence varies substantially worldwide [1]. The highest incidence of gastric cancer is observed in East Asia, Eastern Europe, and the Andean region of South America. Indeed, in the Peruvian population, gastric cancer ranks second in incidence among men and third among women (22.6 and 20 cases per 100,000 males and females respectively), being the type of cancer with the highest mortality. Comparatively, the incidence of gastric cancer in Peru is approximately five times higher than in the United States and twice that observed in Brazil [1].

Recavarren-Arce et al. and Correa proposed a progression model for the development of intestinal-type gastric adenocarcinoma, which consist of a transition from superficial gastritis to metaplasia to dysplasia and finally, gastric adenocarcinoma [2], [3]. A plethora of socio-economic, environmental, and dietary factors modulate this progression and the individual risk of ultimately developing gastric cancer. Chronic infection of the stomach by the bacterium *Helicobacter pylori* leading to chronic inflammation is a major attributable risk factor [4], although less than 2% of *H. pylori* carriers develop gastric cancer [5]. *Helicobacter pylori* diversity also affects the risk of host gastric cancer, and the presence of the bacterial virulence factor cag+ is one of the most relevant risk factors. While this virulence factor has a frequency of ∼60% in European and US populations, it attains more than 90% in the Peruvian population [6]. Also, while Native American individuals from isolated populations are infected by mostly native strains that resemble Asian strains, due to the Pleistocene Asian origin of Native Americans, individuals living in medium and large urban centers, even if they may have a predominant Native American ancestry, are infected by largely European or hybrid strains brought to the Americas after the 15th century. These strains had largely replaced less virulent or vigorous native strains [7], [8].

Poverty also correlates with gastric adenocarcinoma [9] and while elevated consumption of processed or smoked food and salt are risk factors, frequent intake of fresh fruits and vegetables is protective [10], [11], [12], [13]. Human genetic diversity is also relevant [14]. The observed differences in the incidence of gastric cancer worldwide may be due to environmental factors or to the presence of susceptibility genetic variants that are more frequent in populations with high incidence of the disease, but the identification and discrimination of these factors is challenging. Although common susceptibility genetic variants have been identified in European and Chinese populations by genome-wide and candidate-gene association studies in genes such as *PLCE* [15], *IL1B* [14], *IL8* [16], [17], [18], *IL1RN* [19], and *PTGS2* [20], these variants account for a small portion of the genetic variance associated with sporadic gastric cancer.

Peru, with its high incidence of gastric cancer, has the largest Native American population in South America [21] and large cities such as Lima are populated by people classified as mestizo (i.e., individuals with admixture from Africans, Europeans, and Native Americans). If there is a human genetic basis for the high incidence of gastric cancer in the Andean region, we expect the admixed population from Lima to harbor genetic variants accounting for this high incidence, and if these variants were more common in the Native American genetic background of this population, it would be possible to use the genome-wide strategy of admixture mapping to discover these variants. Admixture mapping studies have recently helped to identify variants associated with prostate cancer [22] in African-American populations, but this approach is yet to be fully applied to Latin American or Latino/Hispanic US populations. In this context, the goals of this case-control genetic association study are: (i) to assess the ethnic composition and its related genetic structure of patients attending large hospitals in Lima; (ii) to test if individual Native American, European and African ancestries are risk factors for gastric cancer, controlling for the effect of non-genetic factors (i.e., socioeconomic, nutritional, and clinical). Socioeconomic, dietary, and clinical information was collected for each participant in the study and ancestry was estimated based on 103 ancestry-informative markers (AIMs). We determined that higher Native American individual ancestry is associated with gastric cancer, but that socioeconomic factors associated both with gastric cancer and ethnicity account for this association. Despite the high incidence of gastric cancer among Peruvians with predominantly Native American ancestry, our results do not point to a clear genetic basis for this discrepancy in incidence. Rather, they suggest a predominant role for ethnic-associated socioeconomic and human ecologic factors and disparities in access to health services.

## Results

We recruited individuals attending Gastroenterology Divisions and prescribed for an endoscopy in three large hospitals in Lima (Table S1). Cases were adults referred for endoscopy and whose biopsies were confirmed positive for gastric cancer by histopathological analyses. The control group was composed of individuals whose biopsies were negative for gastric cancer. Individuals with intestinal metaplasia (n = 46) were included as controls on the assumption that this type of metaplasia does not increase the risk of developing gastric cancer [23], [24], [25].

**Figure 1:**
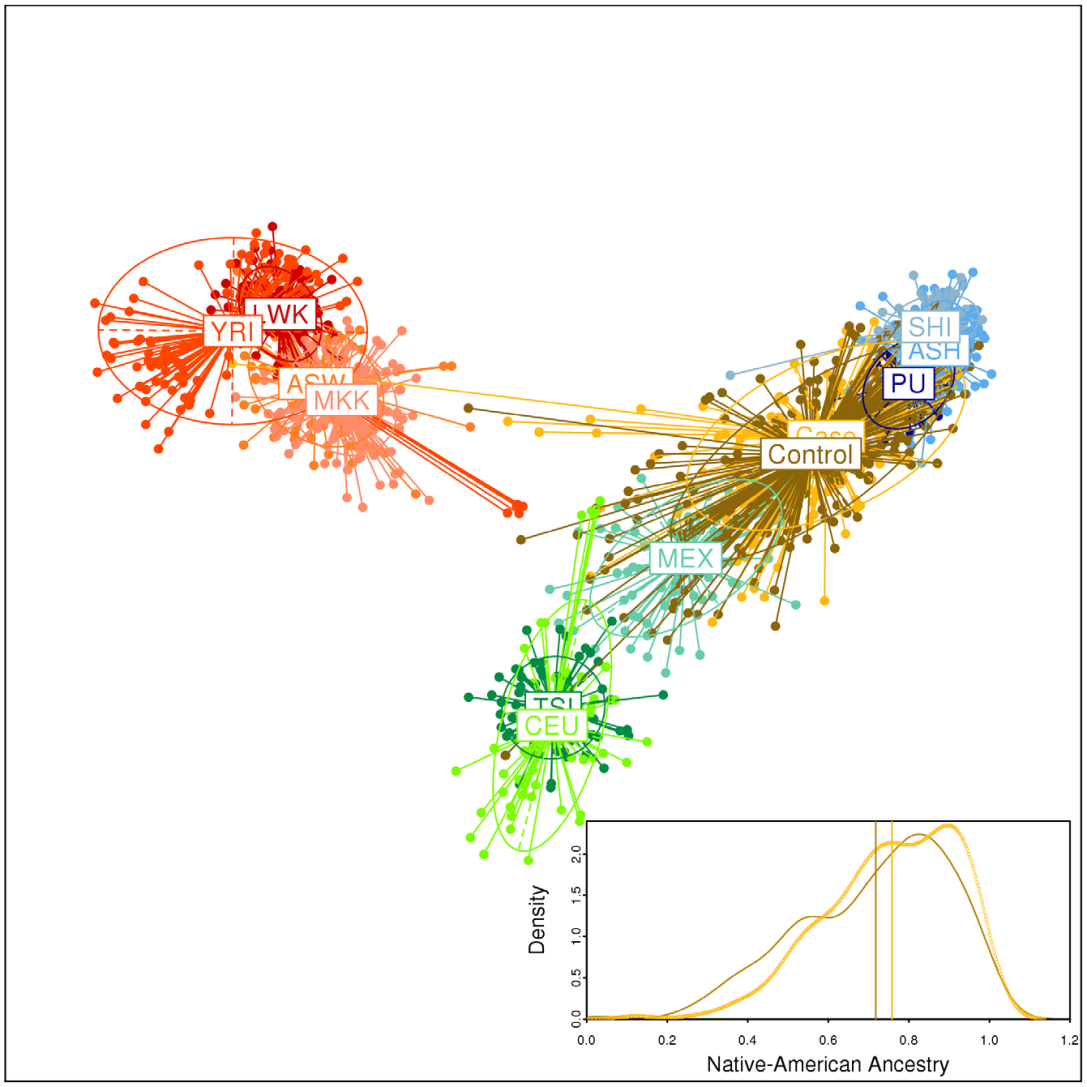
Principal Component Analysis of our Peruvian samples of gastric cancer cases, their controls, and Native Americans in the context of HapMap-III European, African, and Mexican individuals, and distribution of Native American ancestry in cases and controls (**box**)**.** Each individual was genotyped for 103 ancestry informative markers validated by Yaeger et al. (2008). We represent the first (horizontal) and second (vertical) principal components, which capture the 35.3% and 7.7% of total variance, respectively. HapMap individuals: YRI: Yoruba from Nigeria, LWK: Luhya from Kenya, ASW: African American from Southwest USA, MKK: Maasai from Kenya, CEU: Utah residents with European ancestry, TSI: Toscani from Italy, MEX: Mexican ancestry resident in Los Angeles. Peruvian Native Americans: Shimaa (SHI) and Ashaninkas (ASH) from the Matsiguenga ethnic group, and individuals from Puno in the Andes (PU). The box within the figure shows the density plot and means (vertical lines) of Native American ancestry in the gastric cancer cases (yellow) and controls (brown).

We used a validated set of 103 AIMs [26] to estimate Native American, European and Native American individual ancestry for each of the 241 gastric cancer cases and 300 controls recruited for this study. The Principal Component Analysis (PCA, Figure 1) of the individual genotypes for our Peruvian samples (including 296 Native Americans) and for European, African, and Mexican individuals from HapMap-III shows that the AIMs used discriminate between African, European, and Peruvian Native American parental populations, and that the admixed Mexicans (resident in Los Angeles) and the Peruvian gastric cases and controls attending Lima hospitals are placed between Europeans and Native Americans. Moreover, Peruvian gastric cases and controls are relatively closer to the Peruvian Native American parental populations. Although the urban population from Lima is considered as mestizo (i.e., typically admixed), the groups studied herein showed a very high Native American ancestry (78.4% for the cases and 74.6% for the controls, Box in Figure 1), with a low African ancestry (<5%, Figure S1). Interestingly, there is a positive association between Native American ancestry and gastric cancer (logistic regression, OR = 3.69, 95%CI of the OR: 1.34-10.09, p = 0.011, R2 = 0.016), and consequently a negative association with European ancestry.

As expected, variables that are proxies for poverty (low education level, home quality characteristics such as the use of low quality materials, lack of good appliances, and poor sanitary conditions) were associated with gastric cancer (Table 1). Also, some digestive-related symptoms such as burning (p<0.0001), nausea (p<0.0001), vomiting (p<0.0001), and heaviness (p<0.0001) were more frequent in cases than in controls, but these symptoms are likely a consequence of the disease (Table 1). The variable age was not normally distributed (p<0.01, Kolmogorov–Smirnov test), the medians (and deviation interquartile) for controls and cases were 61 (Standard deviation (SD): 21) and 65 (SD: 25) years respectively (p = 0.042, Mann-Whitney test); therefore, this variable was considered as covariate in further logistic regression analyses.

**Table 1:** Socioeconomic, nutritional, and digestive-symptom-related variables and their association with gastric cancer and Native American ancestry.

Variables	P-value of association test with gastric cancer	P-value of association test with Native-American Ancestry
Personal Variables		
Gender	0.0416^b^	0.8621^e^
Ethnicity (self-identification)	0.8692^b^	<0.0001^ e^
Civil status	0.0017^b^	0.0305^f^
Birth in lima	0.3371^b^	0.0007^e^
Socioeconomic Variables		
Education level	0.0013^b^	0.0025^f^
Property of household	0.1241^b^	0.7714^f^
Material of household walls	<0.0001^b^	0.0029^e^
Material of household floor	0.0009^b^	0.0590^e^
Material of household ceiling	<0.0001^b^	0.0023^e^
Type of water supply	0.0007^b^	0.0036^e^
Type of sanitary service	0.0010^b^	<0.0001^e^
Type of garbage collection service	0.0002^b^	0.0056^e^
Fuel used for cooking	0.0149^b^	0.0023^e^
Possession of a refrigerator	<0.0001^b^	0.0020^e^
Possession of a freezer	0.2776^b^	0.5412^e^
Type of energy in the household	0.0057^b^	0.4103^e^
Type of water treatment	0.0069^b^	0.2028^f^
Number of adults in the household	0.0911^c^	0.6643^g^
Number of rooms in the household	0.0665^c^	0.0001^g^
Number of bathroom in the household	0.0009^c^	0.0003^g^
Number of children in the household	0.4894^c^	<0.0001^g^
Number of meals per day	0.9407^c^	0.8532^g^
Number of windows in the household	0.0001^c^	<0.0001^g^
Frequency of eating in a restaurant	0.0690^c^	0.0170^f^
Frequency of eating at the street	0.2345^c^	0.4067^f^
Frequency of eating at home	0.4426^c^	0.9031^f^
Household localization	0.0001^b^	0.0080^f^
Nutritional variables (frequency of consumption of)		
Spicy food	0.8556^c^	0.1903^f^
Steak	0.7363^c^	0.6443^f^
Fish	0.0020^c^	0.5319^f^
Poultry and birds	0.0415^c^	0.5995^f^
Fresh vegetables	0.2226^c^	0.6504^f^
Fresh Fruits	0.0587^c^	0.9235^f^
Tea	0.2864^c^	0.7307^f^
Coffee	0.8658^c^	0.2819^f^
Apple infusion	0.5182^c^	0.0881^f^
Coca leaf infusion	0.3320^c^	0.5237^f^
Symptoms		
Pain	<0.0001^c^	0.7270^f^
Burning	<0.0001^c^	0.4553^f^
Regurgitation	0.0540^c^	0.1220^f^
Nausea	<0.0001^c^	0.0805^f^
Vomit	<0.0001^c^	0.3120^f^
Heaviness	<0.0001^c^	0.2794^f^
Factors from multivariate factor analysis		
Factor 1	0.00002 (OR^a^ 0.68, 95%CI: 0.56–0.80)^d^	0.02017^g^
Factor 2	0.00039 (OR 0.70, 95%CI: 0.58–0.85)^d^	0.00003^g^
Factor 3	<0.0001 (OR 1.8895%CI: 1.54–2.29)^d^	0.0638^g^

Association tests reported in the table are: (^a^) OR: Odd ratio, (^b^): χ^2^ test, (^c^): G-test, (^d^): logistic regression, (^e^): Mann-Withney, (^f^): Kruskal-Wallis, (^g^): Spearman rank order correlation.

We synthesized the wide set of non-genetic variables collected in cases and controls using a multivariate factor analysis, to reduce the dimensionality of these 43 non-genetic variables by capturing the correlation among them (Table 1 and Table S2). Interestingly, the first factor (16.38% of the total variance) is dominated by socioeconomic variables but includes a subset of correlated nutritional variables, higher values of the factor corresponding to wealthier conditions. The second factor (5.95% of total variance) includes subsets of socioeconomic and nutritional variables, as well as complaints, such as pain. The third factor (5.26% of the total variance) is dominated by digestive-related symptoms. We used the individual coordinates for each of these three factors to synthetically represent the original set of 43 variables. Both the first “socioeconomic” factor and the second factor are associated with gastric cancer (OR = 0.68, p = 0.0002 and OR = 0.7, p = 0.0008, respectively) and also with ethnicity (p = 0.02 and p = 0.00003 respectively, where higher values of the factor correspond to better socioeconomic conditions). The third “digestive symptoms” factor is associated with gastric cancer (OR = 2.16, p<0.0001).

The observed association between Native American ancestry and gastric cancer may be due to the effect of confounding socioeconomic or nutritional variables associated with both gastric cancer and ancestry. It is well known that high Native American or African ancestry is associated with poverty in many populations in the Americas (see also [27]). Consistently, we observed that Native American ancestry is associated with variables that are indicators of poverty (many of which were also associated with gastric cancer) as well as their synthetic “socioeconomic” first factor of the multivariate analysis (Table 1). When we controlled for all covariates (i.e., the three factors of the multivariate analysis and age), the association between gastric cancer and Native American ancestry does not persist (OR = 1.28, 95%CI of the OR: 0.37–4.47, p = 0.69). Likewise, when we separately controlled for the effect of socioeconomic conditions (the first factor of the multivariate analysis) or age, the association between gastric cancer and Native American ancestry also does not persist (OR = 2.58, 95%CI of the OR: 0.83–8.07 for factor 1 and OR = 1.01, 95%CI of the OR: 0.99–1.02 for age).

## Discussion

We performed a case-control study in the urban admixed population from Lima (Peru) and determined that Native American individual ancestry is associated with gastric cancer. However, this association seems primarily to be due to the association of socioeconomic variables both with gastric cancer and with Native American ancestry. Consistently, although the association of ancestry with gastric cancer is significant (p = 0.011), ancestry only explains 1.6% of the variance in disease status. When non-genetic covariates are included, their joint effect with ancestry explains 22.3% of the variance in disease status. Therefore, the high incidence of gastric cancer in Peru [1], [28] does not seem to be due to the presence of common susceptibility genetic variants more frequent in Native American populations, but rather to a combination of socioeconomic factors present in this population. However, further studies with larger sample sizes are needed to explore this observation, since the power to detect ancestry genetic effects was limited in the current study. This result is consistent with the relative decrease in gastric cancer incidence in the United States during the last decades, due to the improvement of socioeconomic conditions [29].

Accuracy of ancestry estimations depends on several issues. The first is the number and the nature of markers used to estimate admixture. The 103 AIMs used in this study contain enough information to produce acceptable admixture estimates [26], [30]. Galanter et al. have also showed that a panel of more than 88 AIMs contains enough information to estimate individual admixture with accuracy [31]. A second pervasive methodological issue in estimating admixture is the difficulty in using data from the most representative parental African, European, and Native American populations of the admixed group. In this case, we included as proxy for the parental populations European and African individuals from the HapMap project, and a set of Peruvian Native Americans from the Peruvian Andes and neighboring Eastern areas. While this choice may not be optimal, the 103 SNPs used, being AIMs, mitigated this issue because their frequencies are very different among the parental ethnic groups and highly homogeneous within them [26]. Thus, the use of markers with these characteristics renders our results robust to the choice of suboptimal parental populations. Third, different methods to estimate individual admixture may produce slightly different results even starting out from the same dataset. To test the robustness of our admixture results in respect to the admixture estimation methods, we reanalyzed the data using the alternative maximum-likelihood approaches proposed by Tang et al. [32] and implemented in the software Frappe, and the method by Alexander et al. implemented in the software Admixture v. 1.2 [33]. The three methods produced highly correlated results (Figure S2) and the same pattern of association with gastric cancer (data not shown).

Peruvian individuals born in the countryside have more Native American ancestry on average than do residents of large urban centers (Table 1). In a case-control study, cases may frequently include individuals with more Native American ancestry because they are referred from small countryside health centers to large urban hospitals to receive better healthcare. This referral pattern has less effect on controls, and therefore may create a spurious association of Native American ancestry with disease. However, we recorded places of birth for all study participants, and thereby controlled this potential confounding factor: when we included the place of birth (Lima vs. countryside) as a covariate in the logistic regression, the association of Native American ancestry with gastric cancer persisted, although at a lower significance (p = 0.05 vs. p = 0.011). We conclude that the association between gastric cancer and ancestry is not an artifact of referrals of countryside individuals with high Native American ancestry to our study hospitals. This result emphasizes the importance of gathering birthplace and residence data for genetic association studies with diseases in Latin America, and other regions where most European colonization and admixture occurred in cities, and more autochthonous individuals predominate in rural areas, such as in Melanesia and South Africa.

In this study, we included as controls individuals with intestinal metaplasia (n = 46), assuming that this does not increase the risk of developing gastric cancer [23], [24], [25]. However, this assumption is not universally accepted [34]. When we alternatively assume three ordinal categories of disease risk (i.e., individuals without intestinal metaplasia, with intestinal metaplasia, and with gastric cancer), this progression, assessed by an ordinal logistic regression is also associated with Native American ancestry (OR = 2.83, 95%CI of the OR: 1.10–7.29, p = 0.031), but again, this association does not persist when controlled for all covariates (factor 1, 2, and 3 and age) (OR = 1.08, 95%CI of the OR: 0.35–3.34, p = 0.897). Thus, our results do not depend on the inclusion of intestinal metaplasia individuals as controls.

An issue in our experimental design is that controls were selected as symptomatic individuals attending a gastroenterology service, undergoing an endoscopy and most of them with a gastric lesion: 6 with histologically normal gastric mucosa, 248 with gastritis, and 46 with metaplasia. It could be argued that the optimal control would be composed only by individuals with normal gastric mucosa. However, only through an endoscopy is it possible to accurately ascertain the absence of gastric lesions, and performing an endoscopy for research purposes only, not motivated by gastric-related symptoms is no longer ethically acceptable. On the other hand, using as controls individuals from the general population who did not undergo endoscopy is not necessarily a better choice since this strategy would have included as controls individuals with undetected gastritis [35] and other similar lesions. Therefore, we believe that our controls are the better operational choice for this study, because they are individuals attending the same gastroenterology services as the cases, and since they have undergone an endoscopy, we accurately know the status of their gastric mucosa. Also, in Latin America there is a considerable level of population stratification due to socioeconomic level and ancestry, that are correlated in large urban centers in Latin America (in addition to this study, see Avena et al. 2012 [30] for an example in Buenos Aires and Campbell et al. 2012 [27]). By selecting controls among attendants of the same hospital than cases, we mitigate this other potential source of population stratification between cases and controls.

In this study we report a surprisingly high Native American ancestry (>74%) both in controls and cases attending public hospitals from the now cosmopolitan city of Lima, the national capital that was also the capital of the Spaniard Viceroyalty of Peru for five centuries and therefore, the center of Spaniard colonial power. Large cities in Ecuador, Peru, Bolivia, and Northern Argentina host populations whose usual cultural identification as *mestizo* likely ignores their large Amerindian genetic background. These urban populations are frequently peopled by immigrants from rural areas. Our results suggest that large cities of Western South America host millions of individuals of predominantly Amerindian genetic background. Contemporary international South-to-North migrations from South American cities from the Andean region are also spreading the genetic background of Native Americans worldwide, and it is expected that the almost one million United States immigrants coming from Andean countries [36] have high levels of Native American background. It would not be surprising if these populations, classified as “Hispano/Latino” in the United States, had more Amerindian ancestry than US individuals classified as Native American.

In conclusion, we showed that in the urban admixed population from Lima, Native American individual ancestry is associated with gastric cancer, but this is explained by the association of socioeconomic variables with both gastric cancer and Native American ancestry. Despite the high incidence of gastric cancer in the Peruvian population with a very high Native American ancestry, our result shows that this epidemiological observation does not rely on a genetic basis, suggesting a predominant role for socioeconomic factors and disparities in access to health services. We report a surprisingly high Native American ancestry (>74%) in individuals attending hospitals from the now cosmopolitan city of Lima. Since Native Americans are a neglected group in genomic studies, we suggest that the population from Lima and other large cities in Western South America may be convenient targets of epidemiological studies focused on Native American populations. Pursuit of this avenue of research in subsequently larger studies will begin to close the gap [37] in genetic studies and their potential benefits between European individuals and those from other generally less well served populations.

## Materials and Methods

### Subjects

For this study we initially recruited 576 individuals attending Gastroenterology Divisions of the following three hospitals in Lima, between 2006 and 2009: Arzobispo Loayza, Dos de Mayo (both government-ruled), and the cancer-specialized Instituto Nacional de Enfermedades Neoplásicas. Each participant answered a questionnaire to record age, gender, place of birth and of residence, and other socioeconomic, nutritional, and clinical information (Table 1). Cases were adults referred for endoscopy and whose biopsies were confirmed positive for gastric cancer by histopathological analysis. The control group was also composed of adult individuals referred for endoscopy, but whose biopsies proved negative for gastric cancer. Individuals with premalignant lesions such as dysplasia (n = 3) or with presence of stomach tumors other than gastric cancer (n = 16) were excluded from the study. Individuals with intestinal metaplasia (n = 46) were included as controls (see Discussion). The final number of subjects considered to test in the association study was 241 gastric cancer cases and 300 controls.

We also collected samples from 296 Native American individuals who were used as parental populations to estimate ancestry of the gastric cancer cases and controls. These include 23 farmers from Pichacani (Puno), belonging to the predominant Andean Quechua ethnic group, as well as 87 Shimaa and 186 Ashaninka from the Matsiguenga ethnic group, settled between the Andes and the Amazonian region. For all gastric cancer cases and controls and for the Native Americans, we extracted genomic DNA using the phenol-chlorophorm method described by Sambrook et al. with modifications, or the Gentra Puregene blood kit (Qiagen, USA) [38]. This investigation was approved by IRBs of Asociación Benéfica PRISMA, Universidad Peruana Cayetano Heredia, Johns Hopkins University, Universidade Federal de Minas Gerais, Hospital Arzobispo Loayza, Hospital Dos de Mayo and the Instituto Nacional de Enfermedades Neoplásicas. All participants in the study provided written informed consent.

### Ancestry informative markers (AIMs) and genotyping

To estimate ethnicity for each of the study subjects, we genotyped 106 SNPs that are informative for African, European, and Native American ancestry [26]. The genotyping was performed at the Biomedical Genomic Center of the Children's Hospital Oakland Research Institute (University of Minnesota, MN, USA), using the Sequenom iPLEX platform (San Diego, CA, USA). Briefly, it is based on an allele-specific primer extension followed by separation of alternative alleles by mass spectrometry. The genotyping involved four multiplexed assays, three containing 26 SNPs and one containing 28 SNPs. Before genotyping, DNA samples underwent a Quality Control (QC) procedure that consisted of: (1) a non-allelic quantitative-PCR analysis that measures the quantity of PCR-amplifiable DNA and (2) an end-point reading from a Taqman SNP genotyping assay (Applied Biosystems, Palo Alto, CA, USA) that, in addition to providing a second assessment of the ability of PCR to amplify each sample, is a sensitive indicator of sample-to-sample cross-contamination. After we removed the SNPs rs30125 and rs888861, which showed a call rate <95%, the average call rate for the SNPs was 99.7%. Of the 106 markers, 104 robustly generated call rates for at least 95% of samples, but for the SNP rs2592888 there are no genotypes publicly available for the Hapmap populations and the SNP was excluded from further analyses. Thus, we used genotypes for 103 SNPs to estimate admixture (see Table S3 for the complete list, with their allele frequencies in the study populations).

### Ancestry estimates

We estimated individual ancestry using the following three parental groups composed of unrelated individuals: (1) West African Yoruba from Nigeria (YRI – 118 individuals from the HapMap II/III project); (2) Utah individuals with European ancestry available at the *Centre d'Etude du Polymorphisme Humain-*CEPH collection (CEU – 60 individuals from the HapMap II Project) and (3) 296 Peruvian Native Americans collected by our group. The genotypes for Africans and Europeans were obtained from the public HapMap database [39], while the Native Americans were genotyped for this study.

We estimated the individual ancestry and its 90% credibility interval using the method implemented in the program Structure 2.3.3 [40], [41]. It fits a Bayesian probability model of population structure and admixture using a Markov Chain Monte Carlo (MCMC) procedure, estimating the contribution of K parental populations to the genomes of individuals from the admixed population. We assumed that the HapMap YRI and CEU and our Native American samples were representative of the parental populations and that the gastric cancer cases and controls from Lima were admixed individuals. For this data set, each Structure run had 50,000 burn-in steps followed by 250,000 MCMC steps, and was repeated three times to allow checking for the robustness of the results. This length of the run and the checking procedure exclude the undesirable lack of convergence of the Markov Chains, which happens when the procedure does not properly explore the space of model parameters. All runs were performed assuming three clusters (K = 3), lambda was set to 1.0, and α parameters were estimated for each of the three clusters, GENSBACK  = 2, MIGRPRIOR  = 0.05 and we did not use a priori information for the individuals from parental populations to assist the clustering (USEPOPINFO  = 0).

### Statistical analysis

To represent the genetic structure of our samples in the context of parental population diversity, we performed principal component analysis (PCA) of individual genotypes (Figure 1), as implemented in the software Adegenet and Ade4 for R environment [42], [43]. We also used Ade4 to apply a clustering method that is based on the PCA two-dimensional representation of individuals and their population centroid, designing bi-dimensional ellipses of dispersion. In addition to gender, age and self-reported ethnicity, a wide set of socioeconomic, nutritional, and clinical information was collected including civil status, place of birth, education level, household conditions, eating habits, frequency of consumption of fruits, vegetables, meat and poultry, infusions, as well as gastric-related symptoms. This information was organized in binary, ordinal, or categorical variables, as detailed in Table S4). We excluded variables that had more than 10% missing data, and a final set of 43 variables were included in subsequent analyses. To reduce the dimensionality of this set of personal, socioeconomic, nutritional, and clinical non-genetic variables we performed a multivariate factor analyses using the Statistical Package for the Social Sciences (SPSS) software (SPSS 19 for Windows, SPSS Inc, Chicago, IL, USA). The factor analysis synthesizes the variance of the original set of variables in a specified minor number of transformed variables (in our case 3), called factors. Each factor captures correlated information on the original dataset but the factors are uncorrelated among them [44].

To test the association between these non-genetic variables or its representation obtained by multivariate factor analyses with gastric cancer (a binary trait) and ancestry (a continuous trait), we used the following statistical tests: logistic regression for continuous vs. binary traits (or Ordinal Logistic Regression for continuous vs. an ordinal dependent variable), G-test for ordinal vs. binary traits, χ^2^ test for categorical vs. binary traits, Spearman rank order correlation for continuous vs. continuous traits, and Mann-Withney (2 categories) or Kruskal-Wallis (>2 categories) tests for ordinal vs. continuous traits. These analyses were performed in R environment. Ordinal logistic regression was performed using the ‘rms’ R package [45].

To test the association of ethnicity with gastric cancer we analyzed 443 individuals (245 cases and 198 controls) for whom we have a histopathological diagnosis, collected personal, socioeconomic, dietary data, and estimated ancestry. We used the logistic regression (observing the R^2^ Nagelkerk value – SPSS software) to test the association of ancestry with gastric cancer, which allowed us to control the effect of potential confounding variables (i.e., covariates) that were associated with disease status or ancestry. We included as covariates the original set of non-genetics variables or its factor-analysis synthetic representation.

## Supporting Information

Figure S1 **Barplot of individual ancestry estimated with the software Structure for Africans** (**red**)**, Europeans** (**green**)**, and Native Americans** (**blue**)**, as well as gastric cancer cases and controls.**
(TIF)

Figure S2 **Scatterplot and Spearman correlation between individual Native American ancestry estimates by Structure versus Frappe** (**a**) **and Admixture** (**b**) **methods.** Frappe was run with 100,000 maximum iteration of EM, K = 3 and 10,000 optional convergence threshold. Variations of these parameters did not show differences in results. Admixture was run using the default parameters with K = 3.
(TIF)

Table S1 **Distribution of cases and controls across hospitals and association with Native American ancestry.**
(DOC)

Table S2 **Socioeconomic, nutritional, and digestive-symptom-related variables, their LOD scores with the three first factors of the multivariate factor analysis and significance of Spearman correlation between individual values of the variables and coordinates on each factor.**
(DOCX)

Table S3 **Allele frequencies in the populations included in this study for the 103 Ancestry Informative Markers used in the study.**
(DOC)

Table S4 **Classification of socioeconomic, nutritional, and digestive-symptom-related variables used in** **Table 1** **and their values.**
(DOC)
