# Supplementary figures and images for: Socioeconomic and Nutritional Factors Account for the Association of Gastric Cancer with Amerindian Ancestry in a Latin American Admixed Population
Source: PLoS One. 2012 Aug 3;7(8):e41200. doi: 10.1371/journal.pone.0041200 (PMC3411699; doi:10.1371/journal.pone.0041200)

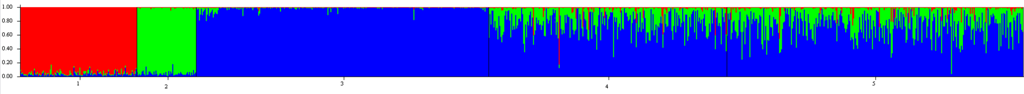

Supplement: Figure S1 — Barplot of individual ancestry estimated with the software Structure for Africans (red), Europeans (green), and Native Americans (blue), as well as gastric cancer cases and controls. (TIF) [file pone.0041200.s001.tif]

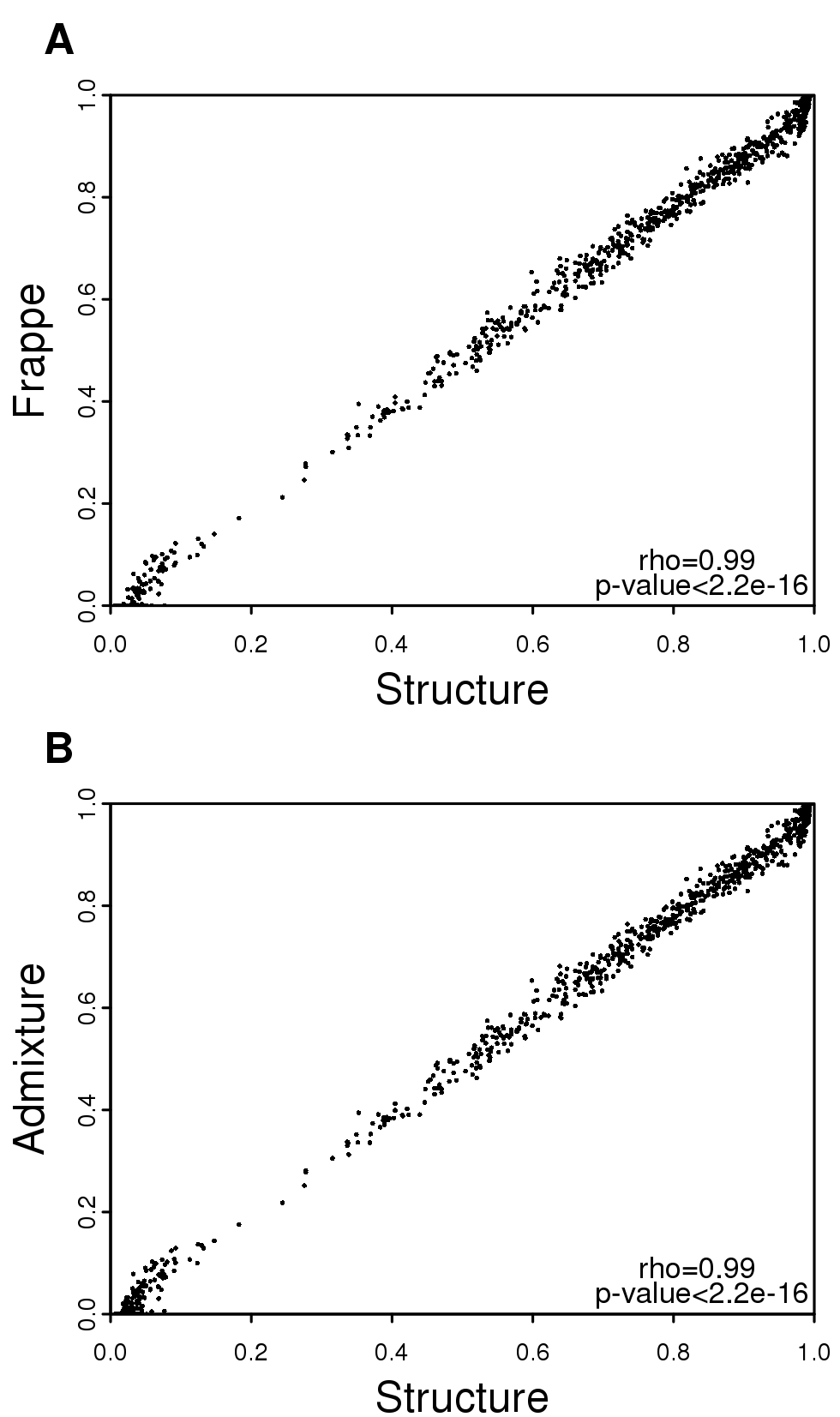

Supplement: Figure S2 — Scatterplot and Spearman correlation between individual Native American ancestry estimates by Structure versus Frappe (a) and Admixture (b) methods. Frappe was run with 100,000 maximum iteration of EM, K = 3 and 10,000 optional convergence threshold. Variations of these parameters did not show differences in results. Admixture was run using the default parameters with K = 3. (TIF) [file pone.0041200.s002.tif]
