# Supplementary material for: Socioeconomic and Nutritional Factors Account for the Association of Gastric Cancer with Amerindian Ancestry in a Latin American Admixed Population
Source: PLoS One. 2012 Aug 3;7(8):e41200. doi: 10.1371/journal.pone.0041200 (PMC3411699; doi:10.1371/journal.pone.0041200)
Supplement: Table S1 — Distribution of cases and controls across hospitals and association with Native American ancestry. (DOC) [file pone.0041200.s003.doc]

Table S1. Distribution of cases and controls across hospitals and its association with Native American ancestry

|  | Hospitals | | |
| --- | --- | --- | --- |
|  | Arzobispo Loayza | Dos de Mayo | Instituto Nacional de Enfermedades Neoplásicas |
| Cases | 108 | 15 | 138 |
| Controls | 139 | 27 | 178 |

We determined that Native American ancestry is not associated with Hospitals for cases or controls (χ^2^ test, P = >0.50).
