# Supplementary material for: Socioeconomic and Nutritional Factors Account for the Association of Gastric Cancer with Amerindian Ancestry in a Latin American Admixed Population
Source: PLoS One. 2012 Aug 3;7(8):e41200. doi: 10.1371/journal.pone.0041200 (PMC3411699; doi:10.1371/journal.pone.0041200)
Supplement: Table S2 — Socioeconomic, nutritional, and digestive-symptom-related variables, their LOD scores with the three first factors of the multivariate factor analysis and significance of Spearman correlation between individual values of the variables and coordinates on each factor. (DOCX) [file pone.0041200.s004.docx]

Table S2. Socioeconomic, nutritional, and digestive-symptom-related variables, their LOD scores with the three first factors of the multivariate factor analysis and significance of Spearman correlation between individual values of the variables and coordinates on each factor.

| Variables | Factor 1 | Factor 2 | Factor 3 |
| --- | --- | --- | --- |
| Personal Variables | | | |
| Gender | -0.029^*^ | -0.173^*^ | -0.027 |
| Ethnicity (self-identification) | -0.144^*^ | -0.060 | 0.126 |
| Civil status | -0.129 | 0.259^*^ | -0.171^*^ |
| Place of birth (Lima vs. countryside) | 0.216^*^ | 0.045 | 0.041^*^ |
| Socioeconomic Variables | | | |
| Education level | 0.444^*^ | -0.042^**^ | 0.262 |
| Property of household | 0.145^*^ | 0.039^*^ | -0.231 |
| Material of household walls | 0.757^**^ | -0.049^*^ | -0.058^*^ |
| Material of household floor | 0.620^**^ | -0.098^*^ | -0.022^*^ |
| Material of household ceiling | 0.726^**^ | -0.063^*^ | -0.041^*^ |
| Type of water supply | 0.641^**^ | 0.026 | -0.128 |
| Type of sanitary service | 0.729^**^ | 0.012^*^ | -0.137^*^ |
| Type of garbage collection service | 0.756^**^ | 0.106 | -0.208 |
| Fuel used for cooking | 0.702^**^ | 0.140^*^ | -0.071 |
| Possession of a refrigerator | 0.751^**^ | 0.109^*^ | -0.069 |
| Possession of a freezer | 0.176 | -0.020^**^ | 0.226^*^ |
| Type of energy in the household | 0.537^**^ | 0.178 | -0.143 |
| Type of water treatment | -0.435^**^ | 0.136^*^ | -0.119^*^ |
| Number of adults in the household | 0.135 | 0.371^**^ | 0.371^*^ |
| Number of rooms in the household | 0.453^*^ | 0.156^**^ | 0.596 |
| Number of bathroom in the household | 0.611^**^ | -0.019^**^ | 0.464^*^ |
| Number of children | -0.017 | 0.240^*^ | 0.110^*^ |
| Number of meals per day | -0.027^*^ | 0.080 | -0.136^*^ |
| Number of windows in the household | 0.528^*^ | -0.067^**^ | 0.546^*^ |
| Frequency of eating in a restaurant | 0.325^*^ | 0.178 | -0.135 |
| Frequency of eating at the street | 0.083 | 0.379 | -0.246 |
| Frequency of eating at home | -0.095 | -0.118^*^ | -0.200 |
| Household localization | 0.523^**^ | -0.081^*^ | 0.077 |
| Nutritional variables (frequency of consumption of) | | | |
| Spicy food | -0.115 | -0.477 | 0.228 |
| Steak | 0.131 | 0.294 | -0.046 |
| Fish | 0.298^*^ | 0.284 | -0.201 |
| Poultry and birds | 0.314^**^ | 0.259 | -0.238 |
| Fresh vegetables | 0.163 | 0.328 | -0.141 |
| Fresh fruits | 0.257^*^ | 0.337 | -0.243 |
| Tea | 0.209^*^ | 0.210 | -0.152 |
| Coffee | -0.021^*^ | 0.338^*^ | 0.062^*^ |
| Apple infusion | -0.020 | 0.080 | -0.031 |
| Coca leaf infusion | -0.151^*^ | 0.085^*^ | 0.115 |
| Symptoms | | | |
| Pain | -0.198 | 0.523^*^ | -0.017^**^ |
| Burning | -0.235 | 0.435^*^ | 0.101^**^ |
| Regurgitation | -0.206 | 0.467 | 0.110^**^ |
| Nausea | -0.409^*^ | 0.365 | 0.339^**^ |
| Vomit | -0.441^*^ | 0.384 | 0.324^**^ |
| Heaviness | -0.229 | 0.397 | 0.176^**^ |

The asterisks denote the P value of the correlation of the individuals factor coordinate and row variables:^*^ P value between 0.05 and 10^-15^ level (2-tailed). ^**^ P value lower than 10^-15^ (2-tailed). Correlation is not significant (P>0.05) if no asterisks are present.
