# Supplementary material for: Socioeconomic and Nutritional Factors Account for the Association of Gastric Cancer with Amerindian Ancestry in a Latin American Admixed Population
Source: PLoS One. 2012 Aug 3;7(8):e41200. doi: 10.1371/journal.pone.0041200 (PMC3411699; doi:10.1371/journal.pone.0041200)
Supplement: Table S3 — Allele frequencies in the populations included in this study for the 103 Ancestry Informative Markers used in the study. (DOC) [file pone.0041200.s005.doc]

Table S3. Allele frequencies in the populations included in this study for the 103 Ancestry Informative Markers used in the study.

| rs | allele | YRI | LWK | ASW | MKK | CEU | TSI | MEX | PU | SHI | ASH | Cases | Controls |
| --- | --- | --- | --- | --- | --- | --- | --- | --- | --- | --- | --- | --- | --- |
| 1004704 | A/G | 0.084 | 0.135 | 0.199 | 0.111 | 0.209 | 0.159 | 0.584 | 0.804 | 0.802 | 0.743 | 0.648 | 0.644 |
| 10131076 | G/A | 0.389 | 0.278 | 0.422 | 0.518 | 0.882 | 0.864 | 0.864 | 1.000 | 1.000 | 1.000 | 0.965 | 0.933 |
| 1013459 | A/G | 0.226 | 0.294 | 0.398 | 0.424 | 0.955 | 0.841 | 0.877 | 1.000 | 1.000 | 1.000 | 0.950 | 0.945 |
| 10214949 | A/G | 0.434 | 0.417 | 0.506 | 0.553 | 0.864 | 0.841 | 0.942 | 0.891 | 0.989 | 0.973 | 0.940 | 0.938 |
| 10248051 | C/T | 0.124 | 0.144 | 0.283 | 0.333 | 0.736 | 0.784 | 0.591 | 0.413 | 0.552 | 0.610 | 0.507 | 0.503 |
| 1036543 | C/T | 0.664 | 0.678 | 0.556 | 0.494 | 0.018 | 0.057 | 0.426 | 0.682 | 0.906 | 0.722 | 0.681 | 0.636 |
| 10484578 | G/A | 0.000 | NA | NA | NA | 0.717 | NA | NA | 0.870 | 0.948 | 0.908 | 0.658 | 0.672 |
| 10486576 | G/A | NA | NA | NA | NA | 0.133 | NA | NA | 0.783 | 0.872 | 0.797 | 0.656 | 0.653 |
| 10488172 | G/T | 0.000 | 0.006 | 0.090 | 0.003 | 0.164 | 0.170 | 0.448 | 0.761 | 0.954 | 0.938 | 0.752 | 0.769 |
| 10491097 | A/G | 0.978 | 0.978 | 0.855 | 0.842 | 0.309 | 0.199 | 0.532 | 0.675 | 0.905 | 0.858 | 0.640 | 0.635 |
| 10491654 | T/C | 0.611 | 0.511 | 0.464 | 0.482 | 0.324 | 0.253 | 0.513 | 0.848 | 0.592 | 0.559 | 0.720 | 0.752 |
| 10492585 | A/G | 0.025 | NA | NA | NA | 0.983 | NA | NA | 1.000 | 0.977 | 0.960 | 0.940 | 0.922 |
| 10497705 | C/T | 0.155 | 0.233 | 0.151 | 0.281 | 0.445 | 0.318 | 0.500 | 0.978 | 0.977 | 0.989 | 0.821 | 0.768 |
| 10498255 | A/G | 0.212 | 0.211 | 0.373 | 0.342 | 0.845 | 0.682 | 0.818 | 1.000 | 1.000 | 0.997 | 0.929 | 0.913 |
| 10498919 | G/C | 1.000 | NA | NA | NA | 1.000 | NA | NA | 0.565 | 0.385 | 0.478 | 0.505 | 0.464 |
| 10500505 | A/T | 0.108 | NA | NA | NA | 0.217 | NA | NA | 0.587 | 0.534 | 0.549 | 0.527 | 0.541 |
| 10501474 | C/T | 0.142 | NA | NA | NA | 0.717 | NA | NA | 0.913 | 0.924 | 0.986 | 0.851 | 0.846 |
| 10506816 | T/A | 0.275 | NA | NA | NA | 0.966 | NA | NA | 0.932 | 1.000 | 0.938 | 0.915 | 0.922 |
| 10507688 | A/G | 1.000 | NA | NA | NA | 0.867 | NA | NA | 0.370 | 0.401 | 0.514 | 0.515 | 0.581 |
| 10508349 | A/G | 0.018 | 0.011 | 0.012 | 0.009 | 0.000 | NA | 0.364 | 0.783 | 0.651 | 0.527 | 0.550 | 0.587 |
| 10510791 | G/C | 0.175 | NA | NA | NA | 0.383 | NA | NA | 0.891 | 0.576 | 0.773 | 0.723 | 0.741 |
| 10515535 | G/A | 0.042 | NA | 0.200 | 0.176 | 0.518 | 0.608 | 0.623 | 0.630 | 0.494 | 0.514 | 0.512 | 0.579 |
| 10515919 | G/A | 0.150 | 0.150 | 0.163 | 0.135 | 0.182 | 0.131 | 0.448 | 0.848 | 0.988 | 0.970 | 0.735 | 0.657 |
| 10517518 | A/G | 0.133 | 0.106 | 0.139 | 0.061 | 0.100 | 0.045 | 0.403 | 0.870 | 0.868 | 0.895 | 0.732 | 0.674 |
| 10519979 | A/G | 0.150 | NA | NA | NA | 0.517 | NA | NA | 0.935 | 1.000 | 1.000 | 0.852 | 0.861 |
| 10520440 | T/G | 0.054 | 0.057 | 0.105 | 0.095 | 0.318 | 0.285 | NA | 0.652 | 0.616 | 0.672 | 0.550 | 0.573 |
| 10520678 | C/T | 0.150 | 0.278 | 0.253 | 0.371 | 0.718 | 0.750 | 0.786 | 1.000 | 0.994 | 1.000 | 0.913 | 0.884 |
| 1073319 | A/G | 0.083 | NA | NA | NA | 0.233 | NA | NA | 0.870 | 0.994 | 0.997 | 0.691 | 0.683 |
| 12953952 | G/A | 0.075 | NA | NA | NA | 0.982 | NA | NA | 1.000 | 1.000 | 1.000 | 0.963 | 0.955 |
| 1353251 | A/G | 0.000 | 0.017 | 0.042 | 0.061 | 0.191 | 0.239 | NA | 0.739 | 0.667 | 0.808 | 0.505 | 0.521 |
| 138022 | G/A | 0.124 | 0.200 | 0.277 | 0.354 | 0.727 | 0.727 | 0.822 | 1.000 | 1.000 | 1.000 | 0.928 | 0.936 |
| 1395771 | G/A | 0.644 | NA | NA | NA | 0.033 | NA | NA | 0.636 | 0.922 | 0.565 | 0.515 | 0.458 |
| 1397618 | A/T | 0.500 | NA | NA | NA | 0.017 | NA | NA | 1.000 | 1.000 | 0.995 | 0.978 | 0.974 |
| 1398829 | A/T | 0.265 | 0.422 | 0.402 | 0.600 | 0.973 | 0.983 | 0.929 | 1.000 | 1.000 | 1.000 | 0.975 | 0.974 |
| 1451928 | G/T | 0.097 | 0.083 | 0.110 | 0.129 | 0.218 | 0.125 | 0.351 | 0.909 | 0.560 | 0.641 | 0.736 | 0.672 |
| 1470524 | T/C | 0.819 | 0.822 | 0.675 | 0.652 | 0.191 | 0.176 | 0.428 | 0.696 | 0.414 | 0.723 | 0.567 | 0.540 |
| 1477277 | C/G | 0.975 | NA | NA | NA | 0.300 | NA | NA | 0.957 | 0.833 | 0.903 | 0.772 | 0.795 |
| 1498991 | G/C | 0.850 | 0.911 | 0.892 | 0.942 | 0.955 | 0.903 | 0.558 | 0.957 | 0.814 | 0.732 | 0.692 | 0.607 |
| 1517634 | G/A | 0.093 | 0.167 | 0.235 | 0.251 | 0.245 | 0.267 | 0.572 | 0.935 | 0.868 | 0.922 | 0.762 | 0.736 |
| 153898 | C/T | 0.914 | 0.883 | NA | 0.683 | 0.264 | 0.256 | 0.558 | 0.957 | 0.965 | 0.889 | 0.858 | 0.794 |
| 1898280 | G/A | 0.853 | 0.903 | 0.679 | 0.765 | 0.118 | 0.125 | 0.405 | 0.761 | 1.000 | 0.965 | 0.644 | 0.600 |
| 1919550 | T/A | 0.000 | NA | NA | NA | 0.033 | NA | NA | 0.909 | 0.891 | 0.957 | 0.706 | 0.689 |
| 1934393 | G/C | 0.783 | 0.767 | 0.651 | 0.544 | 0.173 | 0.284 | 0.442 | 0.870 | 0.408 | 0.535 | 0.676 | 0.628 |
| 1984473 | C/T | 0.063 | 0.133 | 0.181 | 0.327 | 0.655 | 0.591 | 0.697 | 0.891 | 0.937 | 0.970 | 0.857 | 0.843 |
| 1990745 | T/C | 0.049 | 0.006 | 0.102 | 0.041 | 0.148 | 0.188 | 0.435 | 0.957 | 0.698 | 0.776 | 0.704 | 0.667 |
| 2035573 | C/T | 0.050 | NA | NA | NA | 0.267 | NA | NA | 0.804 | 0.767 | 0.772 | 0.644 | 0.597 |
| 2042762 | G/A | 0.000 | NA | NA | NA | 0.033 | NA | NA | 0.587 | 0.931 | 0.710 | 0.512 | 0.450 |
| 2208139 | C/T | 0.155 | 0.133 | 0.217 | 0.176 | 0.400 | 0.386 | 0.592 | 0.891 | 0.773 | 0.984 | 0.841 | 0.848 |
| 2253624 | G/T | NA | NA | NA | NA | 0.983 | NA | NA | 0.978 | 0.977 | 0.844 | 0.930 | 0.907 |
| 2296274 | A/G | 0.018 | 0.073 | 0.151 | 0.221 | 0.773 | 0.744 | 0.825 | 0.935 | 1.000 | 0.973 | 0.903 | 0.877 |
| 249847 | C/T | 0.084 | 0.067 | 0.114 | 0.135 | 0.436 | 0.335 | 0.649 | 0.957 | 1.000 | 0.995 | 0.828 | 0.779 |
| 2569029 | T/G | 0.164 | 0.144 | 0.175 | 0.241 | 0.300 | 0.341 | 0.435 | 0.739 | 0.942 | 0.876 | 0.715 | 0.727 |
| 257748 | A/T | 0.675 | NA | NA | NA | 0.367 | NA | NA | 0.957 | 1.000 | 1.000 | 0.856 | 0.817 |
| 2585901 | T/C | 0.322 | NA | NA | NA | 0.200 | NA | NA | 0.957 | 1.000 | 1.000 | 0.842 | 0.774 |
| 2595456 | G/A | 0.690 | 0.667 | 0.675 | 0.616 | 0.618 | 0.466 | 0.695 | 0.913 | 1.000 | 1.000 | 0.883 | 0.858 |
| 2711070 | G/C | 0.808 | 0.856 | 0.741 | 0.753 | 0.455 | 0.500 | 0.383 | 0.913 | 1.000 | 1.000 | 0.877 | 0.835 |
| 2785279 | T/C | 0.125 | NA | NA | NA | 0.875 | NA | NA | 0.978 | 0.713 | 0.866 | 0.881 | 0.895 |
| 2817611 | G/A | 0.292 | NA | NA | NA | 0.967 | NA | NA | 1.000 | 0.994 | 0.997 | 0.958 | 0.961 |
| 2829454 | G/A | 0.050 | NA | NA | NA | 0.283 | NA | NA | 0.841 | 0.888 | 0.783 | 0.719 | 0.682 |
| 2840290 | C/T | 0.792 | NA | NA | NA | 0.233 | NA | NA | 0.674 | 0.738 | 0.720 | 0.696 | 0.722 |
| 304051 | T/C | 0.179 | 0.283 | 0.323 | 0.442 | 0.682 | 0.636 | 0.877 | 0.978 | 1.000 | 1.000 | 0.906 | 0.880 |
| 354747 | A/G | 0.062 | 0.067 | 0.211 | 0.170 | 0.636 | 0.528 | 0.597 | 0.630 | 0.898 | 0.910 | 0.708 | 0.721 |
| 3768176 | C/G | 0.167 | NA | NA | NA | 0.167 | NA | NA | 0.478 | 0.500 | 0.408 | 0.537 | 0.553 |
| 3806218 | G/A | 0.066 | 0.111 | 0.163 | 0.249 | 0.636 | 0.688 | 0.740 | 0.891 | 0.744 | 0.927 | 0.839 | 0.859 |
| 3828121 | T/C | 1.000 | 0.983 | 0.928 | 0.991 | 0.882 | 0.875 | 0.747 | 0.348 | 0.192 | 0.408 | 0.560 | 0.566 |
| 3860446 | T/C | 0.982 | 0.978 | 0.886 | 0.851 | 0.245 | 0.295 | 0.669 | 0.957 | 1.000 | 0.984 | 0.860 | 0.830 |
| 4013967 | C/T | 0.317 | NA | NA | NA | 0.783 | NA | NA | 1.000 | 1.000 | 0.997 | 0.903 | 0.931 |
| 4034627 | C/T | 0.292 | 0.472 | 0.458 | 0.453 | 0.909 | 0.920 | 0.890 | 0.935 | 0.988 | 0.944 | 0.938 | 0.920 |
| 4076700 | T/C | 0.230 | 0.267 | 0.289 | 0.406 | 0.818 | 0.795 | 0.864 | 1.000 | 1.000 | 0.997 | 0.930 | 0.924 |
| 4130405 | C/A | 0.000 | NA | 0.012 | 0.065 | 0.127 | 0.239 | 0.383 | 0.761 | 0.762 | 0.595 | 0.720 | 0.698 |
| 4130513 | C/T | 0.208 | NA | NA | NA | 0.950 | NA | NA | 0.804 | 0.994 | 0.973 | 0.760 | 0.809 |
| 4625554 | G/A | 0.212 | 0.283 | 0.271 | 0.424 | 0.291 | 0.267 | 0.500 | 0.913 | 0.976 | 0.968 | 0.777 | 0.778 |
| 4657449 | A/G | 0.115 | 0.128 | 0.098 | 0.164 | 0.082 | 0.114 | 0.429 | 0.804 | 0.935 | 0.921 | 0.766 | 0.720 |
| 4733652 | T/C | 0.142 | NA | NA | NA | 0.250 | NA | NA | 0.826 | 0.767 | 0.832 | 0.719 | 0.670 |
| 4762106 | G/A | 0.826 | 0.856 | 0.705 | 0.746 | 0.148 | 0.155 | 0.383 | 0.696 | 0.994 | 0.816 | 0.579 | 0.557 |
| 4852696 | C/G | 0.138 | NA | NA | NA | 0.883 | NA | NA | 0.783 | 0.770 | 0.876 | 0.774 | 0.770 |
| 4934436 | T/C | 0.633 | 0.606 | 0.663 | 0.597 | 0.500 | 0.545 | 0.717 | 0.957 | 0.994 | 0.984 | 0.876 | 0.858 |
| 5000507 | A/T | 0.084 | 0.178 | 0.295 | 0.401 | 0.755 | 0.676 | 0.734 | 1.000 | 0.994 | 1.000 | 0.913 | 0.916 |
| 567992 | A/G | 0.000 | 0.006 | 0.036 | 0.058 | 0.111 | 0.131 | 0.435 | 0.630 | 0.897 | 0.927 | 0.570 | 0.567 |
| 6569792 | A/G | 0.075 | 0.131 | 0.217 | 0.181 | 0.827 | 0.693 | 0.649 | 0.652 | 0.530 | 0.492 | 0.665 | 0.675 |
| 6684063 | G/T | 0.757 | 0.789 | 0.652 | 0.542 | 0.157 | 0.174 | NA | 0.783 | 0.965 | 0.967 | 0.721 | 0.718 |
| 6804094 | T/A | 0.017 | NA | NA | NA | 0.467 | NA | NA | 0.705 | 0.713 | 0.497 | 0.623 | 0.623 |
| 6883095 | A/G | 0.159 | 0.111 | 0.223 | 0.124 | 0.573 | 0.449 | 0.645 | 0.870 | 1.000 | 0.981 | 0.824 | 0.780 |
| 6911727 | T/C | 0.159 | 0.222 | 0.301 | 0.409 | 0.609 | 0.636 | 0.753 | 0.957 | 0.983 | 0.976 | 0.877 | 0.838 |
| 708915 | A/T | 0.750 | NA | NA | NA | 0.167 | NA | NA | 0.659 | 0.865 | 0.789 | 0.535 | 0.533 |
| 719776 | C/G | 0.973 | 0.961 | 0.741 | 0.779 | 0.155 | 0.102 | 0.208 | 0.804 | 0.774 | 0.632 | 0.768 | 0.754 |
| 7463344 | G/C | 0.508 | NA | NA | NA | 1.000 | NA | NA | 1.000 | 1.000 | 1.000 | 0.983 | 0.986 |
| 7535375 | C/T | 0.106 | 0.167 | 0.241 | 0.256 | 0.545 | 0.575 | 0.713 | 0.913 | 0.776 | 0.803 | 0.783 | 0.739 |
| 798887 | G/A | 0.195 | 0.202 | 0.175 | 0.166 | 0.145 | 0.091 | 0.487 | 0.761 | 0.935 | 0.935 | 0.685 | 0.700 |
| 802524 | T/C | 0.173 | 0.233 | 0.343 | 0.363 | 0.991 | 0.943 | 0.916 | 1.000 | 1.000 | 1.000 | 0.975 | 0.966 |
| 842634 | C/T | 0.983 | NA | NA | NA | 0.683 | NA | NA | 0.717 | 0.452 | 0.443 | 0.622 | 0.626 |
| 868179 | G/A | 0.295 | 0.206 | 0.325 | 0.266 | 0.909 | 0.909 | 0.909 | 1.000 | 1.000 | 1.000 | 0.956 | 0.942 |
| 879780 | T/C | 0.181 | 0.343 | 0.392 | 0.386 | 0.918 | 0.926 | 0.838 | 1.000 | 1.000 | 0.997 | 0.938 | 0.947 |
| 9292118 | C/T | 0.235 | 0.344 | 0.398 | 0.541 | 0.682 | 0.790 | 0.799 | 0.978 | 1.000 | 1.000 | 0.915 | 0.917 |
| 9295316 | T/A | 0.017 | NA | NA | NA | 0.133 | NA | NA | 0.761 | 1.000 | 0.995 | 0.786 | 0.720 |
| 9302185 | T/C | 0.107 | 0.152 | 0.226 | 0.319 | 0.800 | 0.824 | 0.908 | 1.000 | 1.000 | 0.978 | 0.938 | 0.931 |
| 9307613 | T/A | 0.127 | NA | NA | NA | 0.517 | NA | NA | 0.955 | 0.837 | 0.951 | 0.873 | 0.843 |
| 9310888 | A/G | 0.465 | 0.500 | 0.572 | 0.629 | 0.909 | 0.983 | 0.942 | 0.978 | 1.000 | 0.995 | 0.968 | 0.962 |
| 9320808 | A/G | 0.863 | 0.861 | 0.783 | 0.725 | 0.155 | 0.182 | 0.532 | 0.696 | 0.965 | 0.901 | 0.746 | 0.707 |
| 9323178 | A/G | 0.115 | 0.117 | 0.177 | 0.279 | 0.555 | 0.454 | 0.655 | 0.913 | 0.954 | 0.992 | 0.835 | 0.788 |
| 9325872 | A/G | 0.814 | 0.843 | 0.705 | 0.804 | 0.318 | 0.375 | 0.630 | 0.978 | 0.994 | 0.997 | 0.889 | 0.857 |
| 948360 | G/A | 0.292 | 0.348 | 0.386 | 0.392 | 0.845 | 0.897 | 0.941 | 1.000 | 1.000 | 0.997 | 0.958 | 0.955 |
| 993314 | C/T | 0.084 | 0.135 | 0.199 | 0.111 | 0.209 | 0.159 | 0.584 | 0.804 | 0.802 | 0.743 | 0.651 | 0.644 |

YRI: Yoruba from Nigeria, LWK: Luhya from Kenya, ASW: African American from South West US, MKK: Maasai from Kenya, CEU: Utah residents with European ancestry, TSI: Toscani from Italy, MEX: Mexican ancestry resident in Los Angeles, PU: individuals from Puno in the Andes, SHI: Shimaa from the Matsiguenga ethnic group, ASH: Ashaninkas from the Matsiguenga ethnic group, NA: Genotyping data not available for this population in the HapMap Project.
