# Supplementary material for: Socioeconomic and Nutritional Factors Account for the Association of Gastric Cancer with Amerindian Ancestry in a Latin American Admixed Population
Source: PLoS One. 2012 Aug 3;7(8):e41200. doi: 10.1371/journal.pone.0041200 (PMC3411699; doi:10.1371/journal.pone.0041200)
Supplement: Table S4 — Classification of socioeconomic, nutritional, and digestive-symptom-related variables used in Table 1 and their values. (DOC) [file pone.0041200.s006.doc]

Table S4. Classification of socioeconomic, nutritional, and digestive-symptom-related variables used in Table 1 and their values used in the statistical analyses.

| Variable | Type | Category name |
| --- | --- | --- |
| Gender | binary | 1- male |
|  |  | 2- female |
| Ethnicity | binary | 1- white |
|  |  | 2- mestizo |
| Civil status | categorical | 1- single |
|  |  | 2- married |
|  |  | 3- widowed |
|  |  | 4- divorced |
|  |  | 5- cohabitant |
| Place of birth (Lima vs. countryside) | binary | 0- no |
|  |  | 1- yes |
| Education level | categorical | 1- no instruction |
|  |  | 2- incomplete elementary |
|  |  | 3- complete elementary |
|  |  | 4- incomplete high school |
|  |  | 5- complete high school |
|  |  | 6- incomplete college |
|  |  | 7- complete college |
|  |  | 8- another |
| Property of household | categorical | 1- own |
|  |  | 2- leased |
|  |  | 3- hosted |
|  |  | 4- hostel |
| Material of household walls | binary | 0- another |
|  |  | 1- cement |
| Material of household floor | binary | 0- another |
|  |  | 1- cement |
| Material of household ceiling | binary | 0- another |
|  |  | 1- cement |
| Type of water supply | binary | 0- another |
|  |  | 1- public supply |
| Type of sanitary service | binary | 0- another |
|  |  | 1- private bathroom |
| Type of garbage collection service | binary | 0- another |
|  |  | 1- public collection |
| Fuel used for cooking | binary | 0- another |
|  |  | 1- gas |
| Possession of a refrigerator | binary | 0- no |
|  |  | 1- yes |
| Possession of a freezer | binary | 0- no |
|  |  | 1- yes |
| Type of energy in the household | binary | 0- another |
|  |  | 1- electric |
| Type of water treatment | categorical | 1- Always |
|  |  | 2- Almost always |
|  |  | 3- Rarely |
|  |  | 4- Never |
| Number of adults in the household | continuous |  |
| Number of rooms in the household | continuous |  |
| Number of bathroom in the household | continuous |  |
| Number of children | continuous |  |
| Number of meal per day | continuous |  |
| Number of windows in the household | continuous |  |
| Frequency of eating in a restaurant | binary | 0- no |
|  |  | 1- yes |
| Frequency of eating at the street | binary | 0- no |
|  |  | 1- yes |
| Frequency of eating at home | binary | 0- no |
|  |  | 1- yes |
| Household localization | categorical | 1- human settlement |
|  |  | 2- rural |
|  |  | 3- urban-marginal |
|  |  | 4- another |
| Frequency of consumption of spicy food | ordinal | 1- Always |
|  |  | 2- Almost always |
|  |  | 3- Rarely |
|  |  | 4- Never |
| Frequency of consumption of steak | ordinal | 1- never |
|  |  | 2- less or equal 3 times/month |
|  |  | 3- 1 to 2 times/week |
|  |  | 4- more than 3 times/week |
| Frequency of consumption of fish | Ordinal | 1- never |
|  |  | 2- less or equal 3 times/month |
|  |  | 3- 1 to 2 times/week |
|  |  | 4- more than 3 times/week |
| Frequency of consumption of poultry and birds | ordinal | 1- never |
|  |  | 2- less or equal 3 times/month |
|  |  | 3- 1 to 2 times/week |
|  |  | 4- more than 3 times/week |
| Frequency of consumption of fresh vegetables | ordinal | 1- never |
|  |  | 2- less or equal 3 times/month |
|  |  | 3- 1 to 2 times/week |
|  |  | 4- more than 3 times/week |
| Frequency of consumption of fresh fruits | ordinal | 1- never |
|  |  | 2- less or equal 3 times/month |
|  |  | 3- 1 to 2 times/week |
|  |  | 4- more than 3 times/week |
| Frequency of consumption of te | ordinal | 1- never |
|  |  | 2- less or equal 3 times/month |
|  |  | 3- 1 to 2 times/week |
|  |  | 4- more than 3 times/week |
| Frequency of consumption of coffee | ordinal | 1- never |
|  |  | 2- less or equal 3 times/month |
|  |  | 3- 1 to 2 times/week |
|  |  | 4- more than 3 times/week |
| Frequency of consumption of apple infusion | ordinal | 1- never |
|  |  | 2- less or equal 3 times/month |
|  |  | 3- 1 to 2 times/week |
|  |  | 4- more than 3 times/week |
| Frequency of consumption of coca leaf infusion | ordinal | 1- never |
|  |  | 2- less or equal 3 times/month |
|  |  | 3- 1 to 2 times/week |
|  |  | 4- more than 3 times/week |
| Frequency of pain | ordinal | 1- never or rarely |
|  |  | 2- sometimes |
|  |  | 3- frequently |
|  |  | 4- every day |
| Frequency of burning | ordinal | 1- never or rarely |
|  |  | 2- sometimes |
|  |  | 3- frequently |
|  |  | 4- every day |
| Frequency of regurgitation | ordinal | 1- never or rarely |
|  |  | 2- sometimes |
|  |  | 3- frequently |
|  |  | 4- every day |
| Frequency of nausea | ordinal | 1- never or rarely |
|  |  | 2- sometimes |
|  |  | 3- frequently |
|  |  | 4- every day |
| Frequency of vomit | ordinal | 1- never or rarely |
|  |  | 2- sometimes |
|  |  | 3- frequently |
|  |  | 4- every day |
| Frequency of heaviness | ordinal | 1- never or rarely |
|  |  | 2- sometimes |
|  |  | 3- frequently |
|  |  | 4- every day |
